# Assessment of desiccants and their instructions for use in rapid diagnostic tests

**DOI:** 10.1186/1475-2875-11-326

**Published:** 2012-09-13

**Authors:** Barbara Barbé, Philippe Gillet, Greet Beelaert, Katrien Fransen, Jan Jacobs

**Affiliations:** 1 Unit of Tropical Laboratory Medicine, Department of Clinical Sciences, Institute of Tropical Medicine, Antwerp, Belgium; 2 HIV/STI Reference Laboratory, Department of Clinical Sciences, Institute of Tropical Medicine, Antwerp, Belgium

**Keywords:** Desiccant, Silica gel, Rapid diagnostic test, RDT-malaria, HIV, Diagnosis

## Abstract

**Background:**

Malaria rapid diagnostic tests (RDTs) are protected from humidity-caused degradation by a desiccant added to the device packaging. The present study assessed malaria RDT products for the availability, type and design of desiccants and their information supplied in the instructions for use (IFU).

**Methods:**

Criteria were based on recommendations of the World Health Organization (WHO), the European Community (CE) and own observations. Silica gel sachets were defined as self-indicating (all beads coated with a humidity indicator that changes colour upon saturation), partial-indicating (part of beads coated) and non-indicating (none of the beads coated). Indicating silica gel sachets were individually assessed for humidity saturation and (in case of partial-indicating silica gels) for the presence of indicating beads.

**Results:**

Fifty malaria RDT products from 25 manufacturers were assessed, 14 (28%) products were listed by the “Global Fund Quality Assurance Policy” and 31 (62%) were CE-marked. All but one product contained a desiccant, mostly (47/50, 94%) silica gel. Twenty (40%) RDT products (one with no desiccant and 19 with non-indicating desiccant) did not meet the WHO guidelines recommending indicating desiccant. All RDT products with self- or partial-indicating silica gel (n = 22 and 8 respectively) contained the toxic cobalt dichloride as humidity indicator. Colour change indicating humidity saturation was observed for 8/16 RDT products, at a median incidence of 0.8% (range 0.05%-4.6%) of sachets inspected. In all RDTs with partial-indicating silica gel, sachets with no colour indicating beads were found (median proportion 13.5% (0.6% - 17.8%) per product) and additional light was needed to assess the humidity colour. Less than half (14/30, 47%) IFUs of RDT products with indicating desiccants mentioned to check the humidity saturation before using the test. Information on properties, safety hazards and disposal of the desiccant was not included in any of the IFUs. There were no differences between Global Fund-listed and CE marked RDT products compared to those which were not. Similar findings were noted for a panel of 11 HIV RDTs that was assessed with the same checklist as the malaria RDTs.

**Conclusion:**

RDTs showed shortcomings in desiccant type and information supplied in the IFU.

## Background

Since 2010, the World Health Organization (WHO) recommends parasitological confirmation of clinical suspicion of malaria before treatment is started. This confirmation can be done either by microscopy or by a malaria rapid diagnostic test (RDT) [1-4].

Malaria RDTs are based on lateral flow immunochromatography, whereby parasite-specific antigens are detected. Their reliability, robustness and simplicity of use have scaled up their supply and procurement worldwide. It is estimated that to date over 200 different RDT products are marketed by 60 manufacturers [1]. The number of malaria RDTs produced annually has increased from 45 million in 2008 to 88 million in 2010 [4].

A major constraint of RDTs is degradation by extreme temperatures and humidity [1,2,5,6]. Therefore, strict temperature control and protection against humidity during transport and storage is necessary. Protection to humidity is assured by packing each individual RDT device (cassette or strip) in a sealed, impermeable pouch containing a desiccant which absorbs humidity [1,7,8].

During laboratory evaluations of RDTs and field projects, staff of the Institute of Tropical Medicine (ITM) noted several problems with the availability, type and manipulation of desiccants in RDTs and previously a number of shortcomings in instructions for use (IFU) of RDTs were studied [9]. Therefore, it was decided to assess a panel of malaria RDT products for the availability, type and design of the desiccants as well as for the completeness and relevance of the information in the IFU.

RDTs based on immunochromatography targeting other pathogens may suffer also from humidity degradation, and ITM staff observed humidity-related problems in Human Immunodefiency Virus (HIV) RDTs [10]. Therefore, an additional panel of HIV RDTs was assessed.

## Methods

### Study design

A panel of RDT products (Additional file 1) was assessed for the desiccant type supplied within the RDT device packaging and for the information mentioned in the IFU. Assessments were performed as part of RDT product panel evaluations and other studies about RDT performance [9,11-17] and at two time points, 2009 and 2012. For some RDT products, more than one lot was assessed. Unless otherwise stated, the results of the first evaluation and first lot assessed are reported and changes over time and differences within lots are presented separately.

### Panel of RDT products

Malaria RDTs marketed as devices consisting of cassettes, cardboard boxes and hybrids (nitrocellulose strips to be dipped into plastic wells) were selected. They were checked for the presence of the CE (European Community) label and evidence of good manufacturing practice (GMP) based on their inclusion in the “List of RDT kits for malaria classified according to the Global Fund Quality Assurance Policy, version 3” of the Global Fund (further shortly referred to as “Global Fund list”) [18]. They included two-, three- and four-band tests, targeting the common Plasmodium antigens. Additional file 1 lists the different RDT products with information about the presence of the CE label and their inclusion in the Global Fund list, as well as the time points of evaluation. It was decided not to display the results of individual RDT products or manufacturers, as desiccants are mostly produced by sub-contractors and may differ from lot to lot.

### Different types of desiccants

**Figure 1:**
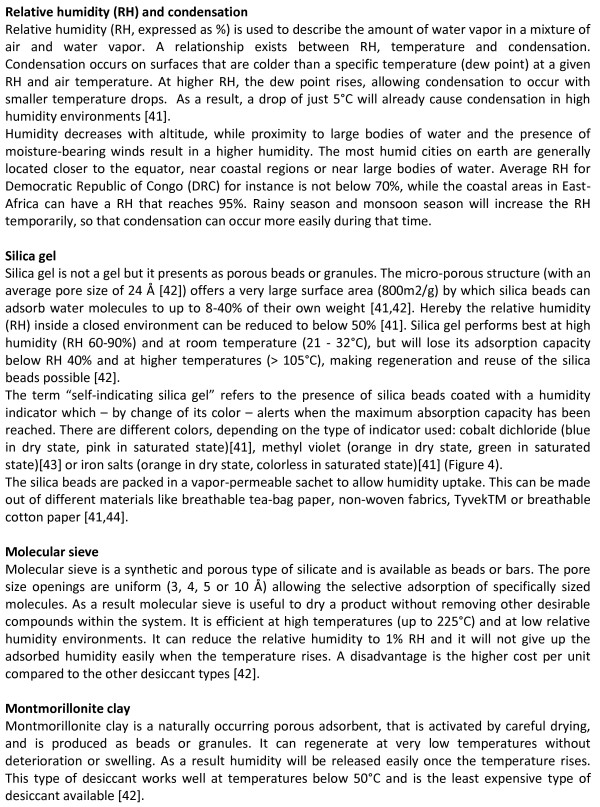
**Background information: relative humidity, condensation and designs of desiccants **[41,42,43,44]**.**

**Figure 2:**
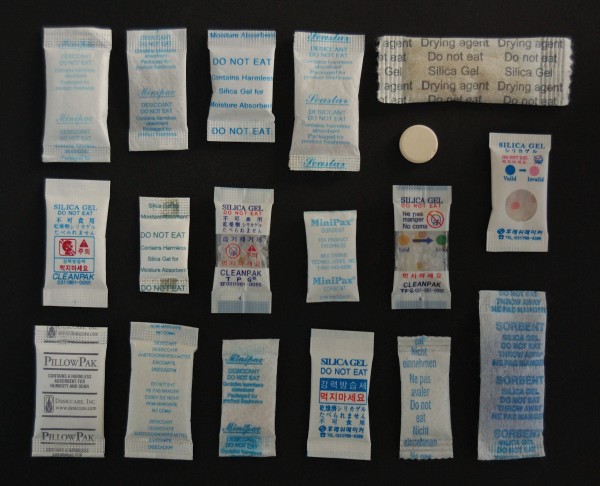
**Examples of desiccants used in RDTs.** Note the tablet (upper right), supplied as desiccant but lacking identification or warning.

Desiccants for in-vitro diagnostic products may include silica gel, molecular sieve or Montmorillonite clay (Figure 1). For RDTs, silica gel is the most frequently used. Unlike its name may suggest, it is not gel but it consists of beads or granules which are packaged in a sachet (Figure 2). This sachet is vapor permeable to allow humidity uptake. Silica gel may be coated with a humidity sensitive indicator which – by change of its colour - indicates when the maximal absorption capacity has been reached and the silica gel is saturated. In industry, silica gel with humidity indicator is referred to as “self-indicating”, in contrast to non-indicating gel. Non-indicating silica gel usually is transparent or opaque while self-indicating silica gel is available in different colours, depending on the type of indicator used (Figure 1). In addition, silica gel sachets in which a majority of non-coloured (non-indicating) beads are mixed with a few indicator (coloured, self-indicating) beads are marketed. For the purpose of this study, such sachets are referred to as “partial-indicating”.

### Assessment of desiccants and instructions for use

A checklist was made to assess desiccants and the IFU. Items were based on recommendations and guidelines about safety and efficacy of the desiccants issued by WHO [1,19-23] and regulatory authorities [24-27], supplemented with criteria derived from bench and field experiences of ITM staff. Table 1 lists the different items, together with the criteria used. The composition of the silica gel humidity indicator was deducted by its colour appearance. To assess the RDTs by the checklist, the device packaging of the different RDT products was opened, the desiccant was inspected and the IFU (limited to the English-part language) was carefully read by two independent readers.

**Table 1:** Desiccant in RDTs

**Items checked**	**Criteria, background and reference**
1. Presence of desiccant in device packaging	Desiccant protects the RDT from humidity before the device packaging is opened [20]
2. Humidity indicator	
2.1. Presence of humidity indicator (self-indicating, partial-indicating, non-indicating (Figure 1)	Desiccant with a colour indicator of humidity is preferred [1]
2.2. Composition of humidity indicator (based on colour appearance)	Cobalt dichloride should be avoided [24,25,27]
3. Material of desiccant sachet:	
3.1. Permeable	Sachet should be vapor permeable to allow humidity uptake
3.2. Transparent	
Colour of beads easily visible with no additional light source needed, additional light source needed, sachet not transparent precluding inspection of colour change	Sachet should be transparent to allow easy colour check of the humidity indicator
4. Warning message on sachet:	
4.1. Not to eat the content of the sachet	The desiccant may be harmful if swallowed, so it should be kept away from children [20]
5. Information available in instructions for use:	
5.1. To check the colour of the silica gel after opening the device packaging	Silica gel colour should be checked upon opening of device packaging [19,21-23]
5.2. How to interpret the colour change of the silica gel	Interpretation of colour indication should be mentioned in IFU or on sachet
5.3. What to do when the silica gel is saturated	The RDT should be discarded and a new RDT should be used [21-23]
5.4. To discard the sachet after colour has been checked	Once the packet is opened, the sachet should be discarded [20]
5.5. Information on properties, safety hazards, remedies and safe disposal of the desiccants should be provided by the manufacturer	It is the responsibility of the manufacturer to provide accurate information on this [1]

Items and criteria used to assess RDT desiccant and their instructions for use.

### Inspection of the individual self-indicating and partial-indicating silica gels sachets

In addition to the checklist, the silica gel sachets with humidity indicator of RDTs were – as part of diagnostic accuracy studies - individually assessed for (i) humidity saturation as indicated by colour change of the indicator and (in case of partial-indicating silica gels) (ii) presence or absence of coloured (indicating) beads. Before opening any device packaging, its integrity was checked.

### Data management and statistical analysis

Data were entered in an Excel sheet (Microsoft Corp., Redmond, WA, U.S.A.). Proportions and 95% confidence intervals (CI) were calculated. Differences between proportions were tested for significance using the Pearson’s Chi-square test or, in case of small sample sizes, a two tailed Fisher’s exact test. A p-value < 0.05 was considered as significant.

### Additional analysis of HIV RDTs

A panel of HIV RDT products that was assessed at ITM as part of the WHO prequalification of diagnostics program was subjected to the same checklist and criteria as used for the malaria RDTs.

## Results

### Panel of RDTs assessed

The RDT panel consisted of 50 products of 25 manufacturers, 14 products (28%) were listed on the Global Fund list and 31 (62%) were CE-marked (Additional file 1). Ten RDT products were assessed both in 2009 and in 2012. Three RDT products were supplied with different types of desiccants (desiccant tablet, non-indicating and partial-indicating silica gel) in different lots.

### Presence, type and content of the desiccant, labelling of sachet

The different desiccant types observed are listed in Table 2. The vast majority (47/50, 94%) of RDTs contained silica gel as a desiccant, supplied as a sachet enclosed in the device packaging. In total, 20 (40%) RDT products (one RDT with no desiccant and 19 with non-indicating desiccant) did not meet the WHO recommendation of adding a self-indicating desiccant to the RDT device packaging. For all eight (16%) partial-indicating silica gels, a strong light source was needed to observe colour change of the indicator through the silica gel sachet.

**Table 2:** Desiccants malaria RDT products (n = 50)

**Desiccant present**	**Type of desiccant**	**Ease of observation of colour change of humidity indicator**	**Warning on desiccant**
Yes (49)	Silica gel, self-indicating (22)	Easily visible (22)	Do not eat (46)
	Silica gel, partial-indicating (8)	Requires additional light (8)	
	Silica gel, non-indicating (17)	NA	
	Tablet unknown identity, non-indicating (2)	NA	No warning
No (1)	NA	NA	NA

Presence and type of desiccant, warning label and ease of observation of colour change of humidity indicator. Data refer to number of RDT products.NA = not applicable.

**Figure 3:**
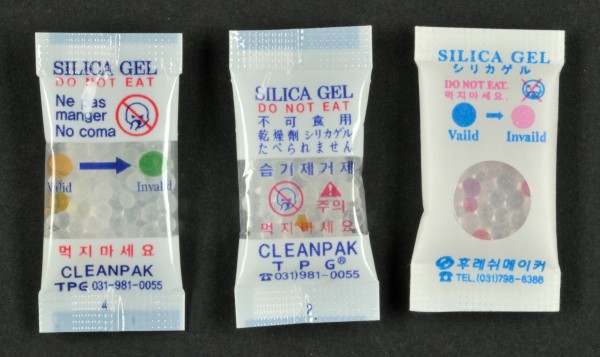
**Examples of three silica gel sachets.** Left: partial-indicating silica gel, with instructions for interpretation. Center: partial-indicating silica gel, no instructions for interpretation. Both sachets (left and center) are packed in a transparent but vapor-impermeable plastic preventing uptake of humidity. Right: vapor-permeable sachet with inspection window. Note warning message written in multiple languages – mandatory in CE-marked products - (sachet at the left) and the pictogram “Do not eat” (all three sachets) which is not clear on the sachet at the right.

On all but one silica gel sachet, the warning “Do not eat” was mentioned. Of note, the tablet supplied as desiccant in two RDT products from one manufacturer showed striking resemblance with a drug tablet and did not contain any identity or warning label (Figure 2). All sachets assessed were made of permeable materials, except for the silica sachets in three products (provided by two manufacturers), which were made out of impermeable plastic (Figure 3). All RDT products with self- or partial-indicating silica gel (n = 30) contained cobalt dichloride as the humidity indicator. Among the 10 RDT products that were assessed both in 2009 and 2012, four had substituted cobalt dichloride by methyl violet (n = 3) or by non-indicating molecular sieve (n = 1).

### Information supplied by the instructions for use

Among the RDT products containing a desiccant with humidity indicator, the IFU of less than half (14/30, 47%) mentioned to check the colour of the silica gel before using the test; their IFU also mentioned how to interpret the colour of the indicator as well as to discard the RDT test in case of humidity saturation. Information on properties, safety hazards, remedies and safe disposal of the desiccant was not included in any of the IFUs.

### Inspection of the individual self-indicating and partial-indicating silica gels sachets

A total of 15,577 silica gel sachets from 16 RDT products were visually inspected immediately after opening the device packaging. They included sachets with self-indicating and partial-indicating silica gels (seven and nine RDT products respectively, Table 3). Three device packages (0.02%) in two RDT products were visibly damaged and excluded from analysis. Colour change indicating humidity saturation (calculated for those sachets with self- and partial-indicating silica beads) was observed for 8/16 RDT products, at a median (range) proportion of 0.8% (0.05% – 4.6%) of sachets inspected. In all RDT products with partial-indicating silica gel, sachets with no colour-indicating beads were observed: the median proportion of sachets containing exclusively non-indicating beads was 13.5%, ranging from 0.6% to 17.8%.

**Table 3:** Silica gel sachets inspected in RDTs: type, presence of indicator and saturation

**N°**	**Number of sachets assessed**	% saturated* (95% CI)	**% without indicator**^**†**^**(95% CI)**
Self-indicating silica gel			
1	625	0.0	
2	1,933	0.05 (0.01 - 2.88)	
3	190	0.0	
4	223	0.0	
5	550	0.0	
6	484	1.2 (0.46 - 2.68)	
7	566	4.6 (3.02 - 6.66)	
Partial-indicating silica gel^‡^			
1	1,507	0.3 (0.08 - 0.75)	9.5 (8.06 - 11.08)
2	2,160	0.1 (0.13 - 0.39)	13.5 (12.06 - 14.99)
3	384	2.5 (1.09 - 4.87)	16.7 (13.08 - 20.78)
4	672	0.2 (0.00 - 0.94)	12.5 (10.09 - 15.24)
5	2,066	0.0	14.6 (13.08 - 16.17)
6	483	0.0	17.8 (14.50 - 21.52)
7	1,721	3.6 (2.75 - 4.68)	10.2 (8.84 - 11.76)
8	483	0.0	0.6 (0.13 - 1.80)
9	146	0.0	6.8 (3.33 - 12.24)

* % saturated: proportion (%) of sachets with humidity indicator that indicated saturation.

^†^ % without indicator: proportion (%) of partial-indicating silica gel sachets in which no indicator (coloured) beads were observed.

^‡^ The number of partial-indicating silica gel sachets exceeds that of table 2 because from one RDT product two lots with different desiccant types, including partial-indicating silica gel, were available.

### Differences between CE marked and global fund listed RDTs and those which were not

There was no differences observed for the assessed criteria between the Global Fund-listed and CE marked RDT products compared to those which were not, except for the warning message “Do not eat” which tended to be more frequently displayed on CE-marked products compared to those which were not (30/31 versus 16/19 respectively, p = 0.053).

### Additional analysis of HIV RDTs

The results of the HIV RDTs are presented in Table 4. A total of 11 products was included, of which six (54%) did not supply a self-indicating desiccant, including one product with no desiccant added to the RDT packaging. All but two desiccants consisted of silica gel, one contained molecular sieve and the other contained Montmorillonite clay. All sachets with partial-indicating silica gel (four products, 36%) needed either a strong light source or opening of the sachet to enable visual inspection of the indicator colour. All desiccant sachets contained the warning message “Do not eat”. Cobalt dichloride was the humidity indicator of choice for three of the five RDT products containing self- and partial-indicating silica gel, while the other two contained silica gel coated either with methyl violet or iron salts. Among the IFU of RDT products supplying self- or partial-indicating silica gel (n = 5, 45%) only one contained instructions to check the indicator colour before use and to discard the RDT when the indicator is saturated.

**Table 4:** Desiccants HIV RDT products (n = 11)

**Desiccant present**	**Type of desiccant**	**Ease of observation of colour change of humidity indicator**	**Warning on desiccant**
Yes (10)	Silica gel, self-indicating (1)	Easily visible (1)	Do not eat (10)
	Silica gel, partial-indicating (4)	Requires additional light (3)	
		Requires opening of sachet (1)	
	Silica gel, non-indicating (3)	NA	
	Other type of desiccant, non-indicating (2)	NA	
No (1)	NA	NA	NA

Presence and type of desiccant, warning label and ease of observation of colour change of humidity indicator. Data refer to number of RDT products.NA = not applicable.

## Discussion

### Summary of findings

The present showed that 40% of 50 malaria RDT products did not meet the WHO recommendation to include a self-indicating desiccant in the device packaging. Silica gel was the most frequently used desiccant. Among the 16 RDT products with indicating silica gel assessed, half contained saturated silica gel sachets in frequencies up to 4.6% of sachets inspected. In all nine RDTs with partial- indicating silica gel, sachets with no colour-indicating beads were found (median 13.5%) and an additional light source was needed to inspect the sachets for colour change of the indicator beads. Cobalt dichloride was the most frequently used humidity indicator. A safety warning was printed on all but three desiccants, but IFU mentioned to check the desiccant for a change in colour in only less than half of RDT products with indicating silica gel and did not contain information about properties, safety hazard and disposal of the desiccant. Similar findings were observed for 11 HIV RDT products.

### Humidity affects RDT performance particularly in tropical settings

It is well known that freezing as well as high temperatures degrade immunochromatographic RDTs and decrease their performance [1]. Likewise, humidity is deleterious [2,5-7]. Humidity weakens the bonds between proteins applied at the control and test lines of the RDT nitrocellulose strip [1] and can complex with sugar molecules thereby delaying particle resolubilization [28]. Humidity results from condensation of water present in the atmosphere; this is especially important in environments with high humidity combined with high temperature (such as in many tropical settings), where a temperature decrease of just 5°C will already cause condensation (Figure 1). A desiccant will reduce the relative humidity inside the device packaging so that condensation will be prevented. WHO recommends to add a self-indicating desiccant to the device packaging for malaria RDTs. Just after opening the device packaging and before using the device, the colour of the humidity indicator should be checked. If it is saturated, the RDT device should not be used but should be discarded [19,21-23].

### The need for a colour-indicating desiccant

In view of the protecting role of the desiccant and the implications of humidity excess, it is striking that 40% of RDT products did not supply a self-indicating desiccant in the device packaging. The fact that, among RDT products with self-indicating gel, up to 4.6% of sachets showed a saturated indicator highlights the relevance of this requirement. The use of non-indicating silica gel does not allow to check the desiccant for saturation (and the device for inappropriateness) and should be discouraged. Further, also sachets with partial-indicating gel did not meet acceptable standards: up to 13.5% of sachets did not contain indicator beads, which points to a problem in production of the sachets. Likewise, the few indicator beads per sachet did not allow to assess colour change through the sachet: to assess colour change, sachets needed to be inspected with an additional light source, which is unpractical and not sustainable in recourse limited settings. Of note, reasons to opt for partial-indicating silica gel are probably cost-related: the estimated whole sale price for a sachet of self-indicating silica gel is 0.70 – 0.95 U.S. dollar cent compared to 0.50 -0.70 cent for a sachet of partial-indicating gel (Byung-Ki Cho, personal communication, March 27 2012). Replacing the sachet material by transparent plastic is unsuccessful as this material does not allow penetration of humidity; in contrast, the use of a paper or tissue sachet with a cellophane reading window meets the requirements (Figure 3).

### Health hazards caused by desiccants

**Figure 4:**
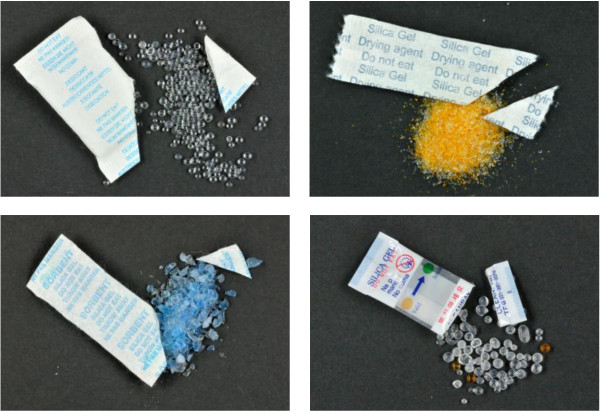
**Different types of silica gel.** Upper left: non-indicating silica gel, transparent or opaque beads. Upper right: self-indicating silica gel, methyl violet as the humidity (colour) indicator. Lower left: self-indicating silica gel, cobalt dichloride. Lower right: partial-indicating silica gel: a majority of non-indicating beads with a few indicator beads.

Cobalt dichloride was by far the most frequently used humidity indicator. Cobalt dichloride has been classified by the EC as a “Carcinogen, category 1B” (H350i: may cause cancer by inhalation) in 2008 and as “Toxic for reproduction, category 1B” (HR60F: may damage fertility) as of 1 December 2012 [24,26,27]. Inhalation of dusts, fumes and mist containing cobalt can cause cancer [24,29]. As an alternative, self-indicating silica gels may be coated with non-toxic and non-flammable indicators such as methyl violet and iron salts (Figure 4). The present study however demonstrates that these non-toxic alternatives are not yet widely implemented. In addition, all desiccants pose a choking hazard when accidently ingested by small children [20,30]. The American Association of Poison Control Centers documented 33,705 desiccant exposures in 2010 of which 89% occurred in children minus six years old [31]. Finally, silica gel can cause irritation to the respiratory system when inhaled, to the digestive tract when ingested and after skin- and eye-contact [32,33].

### Information to be supplied to the end-user: interpretation, safety, waste management

IFU of RDTs should include relevant information about both interpretation and safety of the desiccant. As to interpretation, it was striking that only half of RDT products with indicating desiccant mentioned to check the indicator and to discard the RDT test in case of humidity saturation. Remediation can be easily done by adding appropriate information to the IFU or – such as in the case of the interpretation of the indicator colour – to the desiccant sachet (Figure 3). A field study conducted by QAP (Quality Assurance Project) and WHO in the Philippines and Laos in 2004 showed that omission of checking the humidity indicator of the desiccant was a frequently observed error among end-users, even when it was mentioned in the job aids [19]. Unlike the previous version, the most recent version of the generic job aids for malaria RDTs does not contain depicted information about the check of the humidity indicator [34], and it is suggested to include this information again.

As to safety, it was noted that all but one silica gels sachets mentioned “Do not eat”. The tablets supplied in two RDT products from one manufacturer did not contain information about their nature nor any warning and this is dangerous as they showed misleading similarity with a drug tablet. Further, in the scope of rolling-out of RDTs to remote community settings, one might question universal comprehension of the English text of IFUs. Although pictograms are used (Figure 3), there is no graphical symbol conveying the message “Do not eat” published by the International Organization of Standardization (ISO). As for graphical symbols on in-vitro diagnostics, candidate symbols should be carefully assessed for universal comprehension by end-users of different educational and cultural backgrounds [35].

A final concern is waste management. WHO recommends to discard the sachet immediately after inspection in order to prevent incorrect use or accidental exposure [20] and desiccants are considered as general waste [36].

In the laboratory setting, silica gel sachets can be reused for other applications after regenerating, i.e. evaporation of the absorbed humidity by controlled heating. Examples of such applications include humidity protection of filter paper with Dried Blood Spots or of microscopes during storage and transport [37-40]. Silica gel coated with cobalt dichloride should however not be regenerated, since toxic dust particles may be released during heating [24].

### Limitations and strengths of the study

The present study was cross-sectional and only addressed part of the marketed RDTs products. Further, only in part of RDT products with indicating gels, individual sachets were assessed for the presence of saturated humidity indicators and the absence of indicator beads. In addition, the study was conducted in a temperature- and humidity controlled reference setting and not designed to assess influences of shipment and storage on the saturation of the desiccant.

On the other hand, the selection of RDTs presently studied represented products widely used in the field, including one third of those on the Global Fund list. The results were homogeneous and consistent, with no particular outliers, and they pointed to issues applying to all kind of RDTs based on immunochromatography, as was confirmed by an additional assessment of 11 HIV RDT products.

### Relevance of the findings

The present findings and criteria complement those of a previous study about packaging, labelling and IFU and the proposed operational checklist [9].They further convey messages for manufacturers, regulatory authorities and bulk procurement agencies. As discussed above, manufacturers can easily improve and extent the information added to the IFU. Design and production of desiccants – mostly outsourced to subcontractors – should address issues such as phasing out cobalt dichloride and assuring easy readable humidity indicators. As in the present study, CE and Global Fund-listed RDT products did not score better compared to RDT products that were not, it is clear that there is a role for health authorities in stimulating and monitoring compliance of RDT products with existing requirements. Likewise, suitable and culture-wide warning symbols should be designed. Procurement agencies should include the quality criteria in their product requirements to avoid that cost issues would orient manufacturers to cheaper but inappropriate solutions.

## Conclusion

In conclusion, the design of desiccants currently provided in RDTs should improve, with regard to type (self-indicating desiccant which is easy to inspect), safety (phasing out of cobalt dichloride) and information supplied in the IFU.

## Abbreviations

CE: European Community; CI: Confidence Intervals; DRC: Democratic Republic of Congo; GMP: Good Manufacturing Practice; HIV: Human Immunodeficiency Virus; IFU: Instructions for use; ISO: International organization for standardization; ITM: Institute of Tropical Medicine; QAP: Quality Assurance Project; RDT(s): Rapid diagnostic test(s); RH: Relative Humidity; WHO: World Health Organization.

## Competing interests

The authors declare that they have no competing interests.

## Author’s contributions

BB, PG and JJ designed the study protocol, PG, GB and KF organized sample collection. BB, PG and GB carried out the RDT test evaluations, BB, PG and JJ analyzed and interpreted the results. BB, PG and JJ drafted the manuscript. “All authors critically reviewed the manuscript and approved the final manuscript.”

## Supplementary Material

Additional file 1 List of malaria RDT products that were assessed. Click here for file
